# *Gossypium hirsutum* gene of unknown function, Gohir.A02G039501.1, encodes a potential DNA-binding ALOG protein involved in gene regulation

**DOI:** 10.17912/micropub.biology.000670

**Published:** 2023-09-25

**Authors:** Jonathan Zirkel, Amanda M. Hulse-Kemp, Amanda R. Storm

**Affiliations:** 1 Department of Biology, Western Carolina University, Cullowhee, NC; 2 Genomics and Bioinformatics Research Unit, The Agricultural Research Service of U.S. Department of Agriculture, Raleigh, NC; 3 Department of Crop and Soil Sciences, North Carolina State University, Raleigh, NC

## Abstract

A protein of unknown function encoded by gene Gohir.A02G039501.1 in *Gossypium hirsutum* , was studied using sequence and structure bioinformatic tools leading to its proposed function as a nuclear, DNA-binding ALOG protein involved in gene regulation during organ boundary specification and maintenance. The encoded protein contains a predicted nuclear localization sequence, an ALOG domain with conserved residues in the modeled DNA-binding regions and nearly identical sequence identity to Arabidopsis homologs involved in development of organ boundaries at the shoot apical meristem. The protein was modeled by AlphaFold2 to have a four-helix bundle that is structurally analogous to DNA-binding domains of XerC/D-like recombinases.

**Figure 1. Sequence and Structure Characterization of GhLSH4L-A0A1U8MC48:**
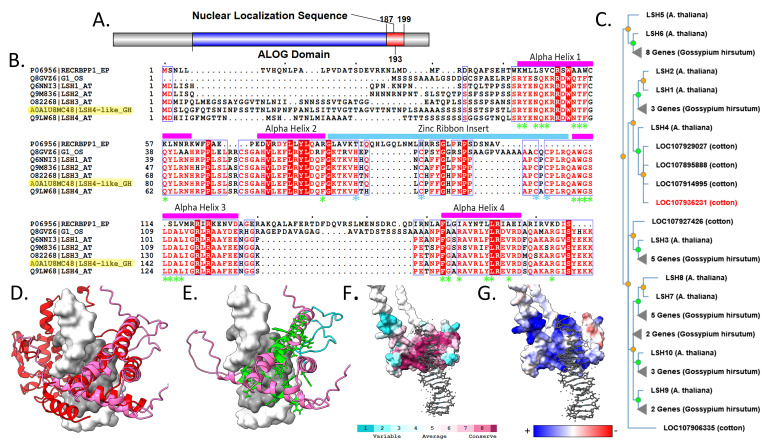
(A) Domain architecture of GhLSH4L-A0A1U8MC48 showing the ALOG domain (blue) and nuclear localization sequence (red), created with IBS (Wenzhong et al., 2015). ALOG domain identified by InterPro, nuclear localization sequence identified by LOCALIZER (Sperschneider et al., 2017). (B) Multi-sequence alignment of CRE recombinase ( *Enterobacteria phage P1* ), G1 ( *Oryza sativa* ), and *Arabidopsis thaliana* LSH1, LSH2, LSH3, LSH4, and *Gossypium hirsutum* GhLSH4L-A0A1U8MC48 (highlighted) created using ClustalOmega (Madeira et al., 2019) and ESPript3 (Robert and Gouet, 2014). Fully conserved residues are highlighted in red while mostly conserved residues are colored red. Predicted ALOG domain alpha helices (capped in magenta), zinc ribbon insert (capped in light blue), DNA binding residues and catalytic residues (green asterisks) are annotated based on the crystal structure of tyrosine recombinase and ALOG proteins (Iyer and Aravind, 2012). Conserved cysteine and histidine residues in the zinc ribbon insert are indicated by a blue asterisks. (C) Phylogenetic tree of *Gossypium hirsutum* and *Arabidopsis thaliana* proteins in the PROTEIN G1-LIKE2 (PTHR31165) family, created using PhyloGenes (Zhang et al., 2020), GhLSH4L-A0A1U8MC48 is highlighted in red. (D) High confidence region of AlphaFold2 (Jumper et al. 2021; Mirdita et al. 2022) structure of GhLSH4L-A0A1U8MC48 (pink) overlaid with CRE recombinase (PDB 1CRX, red) bound to DNA shown in white and grey. (E) GhLSH4L-A0A1U8MC48 structure modeled with bound DNA with potential DNA binding residues (Iyer and Aravind, 2012) shown in green stick representation and Zinc-Ribbon insert shown in blue ribbon (F) GhLSH4L-A0A1U8MC48 structure modeled with bound DNA, showing conserved residues as determined by ConSurf. (Ashkenazy et al., 2016). Teal corresponds to areas that are more variable, and magenta corresponds to areas that are more conserved. Amino acids 28-60 were removed for easier viewing. (G) GhLSH4L-A0A1U8MC48 structure modeled with bound DNA, showing electrostatic potentials as determined by ChimeraX. Amino acids 28-60 were removed for easier viewing. Blue corresponds to areas with positive charge and red shows negative charge. DNA is shown in gray. All structures imaged using ChimeraX (Version 1.3, Pettersen et al., 2021).

## Description

The genomic sequencing of five *Gossypium* allopolyploid species (Chen et al., 2020) revealed thousands of uncharacterized proteins shared across all five species. Proteins within this set of sequences that were deemed favorable for bioinformatic analysis by initial screening were studied individually by students as part of a research course to gain clues to potential function. One of those uncharacterized proteins encoded by the gene LOC107943309 (Gohir.A02G039501_UTX-TM1_v2.1) in upland cotton G *ossypium hirsutum* genome (L. accession Texas Marker-1 (TM-1) version 2.0, annotation version 2.1) was termed ‘light-dependent short hypocotyls 4-like’ (NCBI: XP_016732548, UniProt: A0A1U8MC48). Here we present bioinformatic evidence that this protein is a part of the ALOG ( A rabidopsis L SH1 (light-dependent short hypocotyl) and O ryza G 1) family and contains the necessary sequence and structure for localization to the nucleus and subsequent DNA-binding. This protein will be referred to here as GhLSH4L-A0A1U8MC48 ( *Gossypium hirsutum* light-dependent short hypocotyls 4-like - UniProt ID) as a temporary reference until the protein is studied experimentally.

The ALOG family is specific to eukaryotes, mostly in Streptophytes with over 80% of the family belonging to Magnoliopsida (dicots) although there are distant homologs in some marine metazoan lineages (Iyer and Aravind, 2012) . The precise function of the ALOG family is unknown; however, several studies have been conducted on some family members. Arabidopsis LSH1 conferred a hypersensitive response to certain light conditions when analyzed using photoreceptor mutant backgrounds, additionally LSH1 appeared to mediate seedling development (Zhao et al., 2004) . LSH4 and LSH3 in Arabidopsis were shown to be expressed in boundary cells of shoot organs and regulated by the transcription factors CUC1 and CUC2 (Takeda et al., 2011) . In *Oryza sativa* and *Arabidopsis thaliana* , it has been shown that disruption of certain ALOG domains result in defects with floral development (Cho et al., 2010) . Another *Oryza sativa* homolog, ALOG protein TH1 (TriangularHull1), was shown to be a homodimer via a yeast two-hybrid assay and may function as a transcription repressor regulating cell expansion during lateral development (Peng et al., 2017) .

There is evidence that the ALOG domain was derived from the N-terminal DNA-binding domain (DBD) of the XerC/D-like recombinases, a type of viral retroposon (Iyer and Aravind, 2012) . The N-terminal DBD of CRE recombinase, a tyrosine recombinase, has been experimentally shown to bind to DNA (Guo et al., 1997) through a core four helix-bundle with hydrophobic residues that is also present within the ALOG domain. However, ALOG domains are set apart by a Zinc-Ribbon inserted between helices 2 and 3 (Iyer and Aravind, 2012) . The ALOG domain contains three major groove-contacting helices with a fourth helix located near the C-terminal. This structure is consistent with the XerC/D-like clade (containing recombinases, phage integrases, and integron integrases) which also bind to DNA (Iyer and Aravind, 2012) .

Sequence Features

InterPro (Blum et al., 2020) identified the 260 amino acid protein GhLSH4L-A0A1U8MC48 as a member of the ALOG family (IPR040222), containing the 125-residue domain DUF640 (PF04852) also known as ALOG domain (IPR006936). Sequence analysis by LOCALIZER (Sperschneider et al., 2017) identified a Nuclear Localization Sequence (NLS) near the C terminus. This is supported by Y-Loc, Plant-mPLoc, and Plant-mSubP, which predict GhLSH4L-A0A1U8MC48 localizes to the nucleus similarly to other ALOG proteins such as TH1 that has been observed to localize to the nucleus (Peng et al., 2017) . These features are visualized in a domain architecture ( **Figure 1A** ).

A multi-sequence alignment (MSA) was made using several orthologs of GhLSH4L-A0A1U8MC48 ( **Figure 1B** ) that have been studied experimentally and computationally (Takeda et al., 2011; Iyer and Aravind, 2012; Yoshida et al., 2009; Zhao et al., 2004) , including the closest homologs in Arabidopsis (LSH1-4) and rice (G1) as well as the structural analog viral CRE recombinase (PDB 1CRX). The regions identified as forming the four helix-bundle in homologs (Iyer and Aravind, 2012) are annotated and this structural feature is conserved between the orthologs. This arrangement of helices is highly conserved in ALOG domains and serves as the structural feature needed for the protein to interact with DNA based on structures of the DBD of CRE recombinase where many predicted DNA contacting residues (green asterisks) are located inside of the helices (Iyer and Aravind, 2012) .

A main structural difference between the ALOG family and DBD of tyrosine recombinases is the Zinc-Ribbon (ZnR) insert between helices 2 and 3. The positioning of this region and the presence of conserved positively charged residues suggest this region is involved with ALOG-domain specific DNA contacts (Iyer and Aravind, 2012) . This region is labeled in the MSA (blue bar) and all ALOG proteins contain conserved cysteine and histidine residues (marked with blue asterisks) in characteristic motifs ‘HxxxC’ and ‘CxC’, which support a zinc binding role for this region ( **Figure 1B** ). The full ConSurf results are available as Extended Data.

Homology

PhyloGenes (Zhang et al., 2020) identified GhLSH4L-A0A1U8MC48 as belonging to the PROTEIN G1-LIKE2 family with LSH1-10 in Arabidopsis as orthologs ( **Figure 1C** ). The PhyloGenes pre-computed phylogenetic tree based on the PANTHER family (PTHR31165) shows GhLSH4L-A0A1U8MC48 clusters most closely to LSH4 of *Arabidopsis thaliana* and more distantly to LSH1 and 2, whereas LSH3, LSH5/6, and LSH7-10 appear to form separate clades. Pairwise alignment in BLASTp between GhLSH4L-A0A1U8MC48 and LSH4 in *Arabidopsis thaliana* confirmed that they are close homologs of each other with 89% identity across a 66% Query cover. The MSA shows the similarity between GhLSH4L-A0A1U8MC48 and Arabidopsis LSH4 which differ in only a few residues within the domain region, outside of the variable N-terminal 40 amino acids ( **Figure 1B** ). LSH3 and LSH4 are closely related and have been studied together in Arabidopsis and shown to be activated by transcription factor CUC1, which plays a central role in establishing shoot organ boundaries in embryo development in *Arabidopsis thaliana* . (Takeda et al., 2011) .

Structure Features

AlphaFold2 was used to predict a structure model for GhLSH4L-A0A1U8MC48 ( **Figure 1D, E, F, G** ). The AlphaFold2 model had a high level of confidence in the ALOG domain region from amino acid 55 to 193, which consists of the four helix-bundle as seen in structures of other ALOG proteins (Iyer and Aravind, 2012) . The structure was overlaid with the CRE recombinase N-terminal DNA-Binding domain (PDB: 1CRX) ( **Figure 1D** ) using UCSF ChimeraX MatchMaker. CRE recombinase is a known DNA binding protein (Guo et al., 1997) and was one of the top structural analogs for GhLSH4L-A0A1U8MC48 obtained through DALI (Z score 8.3, rmsd 6.3) (Holm, 2022) . This overlay allowed DNA to be modeled onto the structure of GhLSH4L-A0A1U8MC48 where helices 1 and 3 are positioned to form contacts deep in the major groove. The position of previously identified DNA binding residues in structural analogs (Iyer and Aravind, 2012) , shown in the MSA ( **Figure 1B** , green asterisks), were highlighted in the structure ( **Figure 1E** ) and shown to cluster around the modeled DNA binding site. Although there is very little conservation with the CRE recombinase sequence, all these predicted DNA binding residues were highly conserved between the plant orthologs ( **Figure 1B** ) suggesting that they have a distinct DNA recognition site. ConSurf (Ashkenazy et al., 2016) analysis of the conservation of these residues in GhLSH4L-A0A1U8MC48 showed that they were all highly conserved and cluster around the proposed DNA binding site ( **Figure 1F** ). The UCSF ChimeraX electrostatic potential tool showed this center crevice around the binding site was also highly positive where these residues could interact with the negatively charged DNA backbone ( **Figure 1G** ).

The evidence presented here in the sequence, structure, and homology analysis of GhLSH4L-A0A1U8MC48 support the conclusion that this protein localizes to the nucleus and binds to DNA to function as a transcription factor or recruiter of chromatin remodeling. This is supported by the conservation of predicted DNA binding residues, electrostatic surface potentials, structural similarity to DBD of recombinases, and predicted subcellular localization. The placement of this protein into the ALOG family is further supported by the high sequence similarity and conservation with other ALOG domain proteins as well as phylogenetic evidence.

## Extended Data

Description: ConSurf sequence conservation results for A0A1U8MC48. Resource Type: Dataset. DOI: 10.22002/xm3mf-twv31
